# Plant-Mediated Synthesis of Magnetite Nanoparticles with *Matricaria chamomilla* Aqueous Extract

**DOI:** 10.3390/nano14080729

**Published:** 2024-04-22

**Authors:** Andrea Paut, Lucija Guć, Martina Vrankić, Doris Crnčević, Pavla Šenjug, Damir Pajić, Renata Odžak, Matilda Šprung, Kristian Nakić, Marijan Marciuš, Ante Prkić, Ivana Mitar

**Affiliations:** 1 Faculty of Chemistry and Technology, University of Split, Ruđera Boškovića 35, 21000 Split, Croatia; andrea.paut@ktf-split.hr (A.P.); prkic@ktf-split.hr (A.P.); 2 Faculty of Science, University of Split, Ruđera Boškovića 33, 21000 Split, Croatia; lguc@pmfst.hr (L.G.); dcrncevic@pmfst.hr (D.C.); rodzak@pmfst.hr (R.O.); msprung@pmfst.hr (M.Š.); knakic@pmfst.hr (K.N.); 3 Laboratory for Synthesis and Crystallography of Functional Materials, Division of Materials Physics, Ruđer Bošković Institute, Bijenička 54, 10000 Zagreb, Croatia; martina.vrankic@irb.hr; 4 Department of Physics, Faculty of Science, University of Zagreb, Bijenička Cesta 32, 10000 Zagreb, Croatia; psenjug@phy.hr (P.Š.); dpajic@phy.hr (D.P.); 5 Laboratory for Synthesis of New Materials, Division of Materials Chemistry, Ruđer Bošković Institute, Bijenička 54, 10000 Zagreb, Croatia; marijan.marcius@irb.hr

**Keywords:** green synthesis, magnetite, *Matricaria chamomilla* aqueous extract, biocompatibility, plant magnetic nanoparticle biogenesis

## Abstract

Magnetite nanoparticles (NPs) possess properties that make them suitable for a wide range of applications. In recent years, interest in the synthesis of magnetite NPs and their surface functionalization has increased significantly, especially regarding their application in biomedicine such as for controlled and targeted drug delivery. There are several conventional methods for preparing magnetite NPs, all of which mostly utilize Fe(iii) and Fe(ii) salt precursors. In this study, we present a microwave hydrothermal synthesis for the precipitation of magnetite NPs at temperatures of 200 °C for 20 min and 260 °C for 5 min, with only iron(iii) as a precursor utilizing chamomile flower extract as a stabilizing, capping, and reducing agent. Products were characterized using FTIR, PXRD, SEM, and magnetometry. Our analysis revealed significant differences in the properties of magnetite NPs prepared with this approach, and the conventional two-precursor hydrothermal microwave method (sample MagH). FTIR and PXRD analyses confirmed coated magnetite particles. The temperature and magnetic-field dependence of magnetization indicate their superparamagnetic behavior. Importantly, the results of our study show the noticeable cytotoxicity of coated magnetite NPs—toxic to carcinoma cells but harmless to healthy cells—further emphasizing the potential of these NPs for biomedical applications.

## 1. Introduction

The synthesis of nanoparticles (NPs), especially magnetic NPs, consisting of ferromagnetic materials such as iron oxides, including magnetite (Fe_3_O_4_) and maghemite (γ-Fe_2_O_3_), has experienced remarkable growth in the fields of nanotechnology, materials science, and engineering. Magnetite stands out among all naturally occurring minerals on Earth due to its extraordinary magnetism. Unlike other iron oxides, magnetite contains both Fe^2+^ and Fe^3+^ ions. Its structure is a cubic inverse spinel consisting of a cubic close-packed arrangement of oxide ions, with the tetrahedral sites occupied by Fe^3+^ cations surrounded by four oxygen atoms, while the octahedral sites are occupied by Fe^2+^ and Fe^3+^ ions surrounded by six oxygen atoms so that Fe^3+^ ions are present in both the tetrahedral and octahedral sites. Nano-sized magnetite particles have exceptional physical, chemical, and optical properties that have made them a popular choice for current nanoparticle research. These properties, including high availability, versatility, superparamagnetic behavior, and a large surface-to-volume ratio, enable more efficient binding with various molecules [1,2,3,4,5,6,7]. The superparamagnetic behavior allows for potential manipulation from an external magnetic field, which opens up a wide range of magnetite NP applications such as heavy metal removal [8,9]. Additionally, due to potential biocompatibility, there is also the possibility for broad biomedical applications, for example, targeted drug delivery, bioimaging, hyperthermia therapy, photoablation therapy, biosensors, and theranostics [10,11,12].

Today, much attention is paid to the improvement of various synthesis methods for magnetite NPs. In recent decades, various methods have been developed for the preparation of iron oxide NPs, taking into account the desired properties for the intended applications. Different synthesis approaches such as the co-precipitation, thermal decomposition, microemulsion, sol–gel, and hydrothermal methods are used to control the nucleation and growth of iron oxide NPs from precursor solutions. In these methods, parameters such as the selection and concentration (or molar ratio) of precursors, pressure, reaction temperature and time, pH, and ionic strength are manipulated to achieve precise control of the synthesis process. This emphasis stems from the realization that even minor changes in the production process can have a significant impact on the resulting physical and chemical properties of the particles. For instance, temperature and reaction time play a crucial role in particle size, with the process occurring in two distinct steps: nucleation and crystal growth [10,13]. Therefore, understanding the complex relationship between the synthetic parameters and the properties of NPs is crucial for the design of NPs tailored to specific applications [3,4,14,15].

One of the simplest methods for producing magnetite NP is co-precipitation, in which Fe^2+^ and Fe^3+^ ions are precipitated simultaneously in a basic aqueous environment. Under oxygen-free conditions and a 2:1 molar ratio of Fe^3+^ and Fe^2+^ cations from aqueous iron salt precursor solutions, the complete precipitation of Fe_3_O_4_ typically occurs in a pH range from 9 to 14. However, the size, morphology, and magnetic properties of magnetite NPs synthesized via the co-precipitation method can vary significantly [10,11,16]. On the other hand, microwave-assisted methods, using a professional microwave reactor can precisely control the experimental parameters of the synthesis, thus enabling the fast optimization of the synthesis pathway to produce particles with the desired sizes and shapes [17]. Nevertheless, the major drawback of these methods is the aggregation of the particles. To overcome this issue, researchers have explored the use of various surface modifiers as stabilizing agents, e.g., polyethylene glycol (PEG), ionic liquids, silica, etc., to prevent aggregation and maintain the chemical and physical stability of the NPs. Besides aggregation, the size and lack of biocompatibility are the main problems with magnetite NPs in medical applications. The small size of NPs allows them to penetrate physical barriers and come into contact with intracellular compartments such as the cytoplasm and nucleus. This direct interaction can lead to cell damage or destruction and poses a threat to living organisms. To address these concerns, the surface of the NPs can be modified through the careful selection of an appropriate capping agent to reduce potential toxic effects. Capping agents improve biocompatibility by forming a coating on the surface of the NPs and acting as a protective layer that prevents direct contact between the NPs and cellular components. This minimizes the risk of cell damage, which is critical for the aforementioned medical applications. By carefully selecting a suitable stabilizing and capping agent, the desired properties of the NPs can be well controlled and manipulated. However, the intensive development of the production and application of NPs has raised some concerns about the possible toxic effects on the environment and human and animal health. Various organic solvents, reducing and capping agents, and stabilizers are not only expensive but are also known to be harmful to the environment and have been associated with cytotoxicity and carcinogenicity [18]. Therefore, there is a growing need to develop more environmentally friendly and cost-effective production methods. One way to overcome these challenges is to modify nanoparticle synthesis processes through the careful selection of precursors and the replacement of toxic chemicals with biological materials—green chemistry approaches. In the context of green chemistry, the use of harmless solvents and non-toxic reducing agents is crucial, as this can significantly reduce the overall toxicity of the synthesis process. These agents should be able to effectively stabilize the NPs and be environmentally benign [10,18,19]. To this purpose, various biological materials have been used, including aqueous plant extracts, bacteria, and algae, which have received considerable attention in the synthesis of NPs. Studies have shown that phytochemicals can improve the chemical stability and biocompatibility of NPs by forming a coating on their surface, thus preventing the aggregation of the particles. This coating allows binding with various molecules, thus making the NPs biocompatible and chemically stable [12,20].

Among these options, the use of plant-based synthesis methods offers the simplest and most advantageous approach. Whole organs, tissues, or aqueous extracts of various plant parts, such as seeds, leaves, bark, roots, and fruits can be used in green synthesis. Plants contain a variety of chemicals, including antioxidants, sugars, amino acids, carboxylic acids (citric acid), flavonoids, phenolic compounds, terpenoids, polysaccharides, and tannins, which could play an important role as reducing agents for metal ions, as well as stabilizers and capping agents. Numerous green, plant-based synthesis methods with the utilization of various plant extracts have been documented in the literature. For example, magnetite NPs have been successfully synthesized using biomaterials such as *Dolichos lablab* extract, *Musa acuminata* peel ash, *Cynara carduncculus* leaf extract, Quince (*Cydonia oblonga* Miller) seed extract, Aloe vera and Flaxseed extract, *Kappaphycus alvarezii* seaweed, the natural rubber latex of *Hevea brasiliensis*, lemon juice, *Parkia speciosa* Hassk. pod extract, the plant root extract of *Chromolaena odorata*, *Artemisia tilesii* L. “hairy” roots, Calotropis *procera* aqueous leaf extract, and *Ficus Benghalensis* plant leaf extracts [13,21,22,23,24,25,26,27,28,29,30,31,32]. As already mentioned, plant substances can have multiple functions, so that no additional agents are needed. Notable examples of the green synthesis of iron oxide NPs are using *Mimosa pudica* root extract, onion peel extract and corn silk extract, leaves extract of *Euphorbia helioscopia, Lagenaria siceraria* leaf extract, stevia extract, fruit extracts, and green tea extract in the role of reducing agent and solvent [33,34,35,36,37,38,39,40]. Synthesized plant-based NPs have shown several remarkable properties, such as antibacterial, antioxidant, and anticancer activity, which make them highly desirable for biomedical applications as well as for the production of low-cost adsorbents for water treatment. One such example is the work of Sari et al. in which magnetite particles were synthesized from an aqueous extract of *Graptophyllum pictum* leaves. This extract contains alkaloids and flavonoids, which played multiple crucial roles in the synthesis process: the alkaloids served as a base source and acted as a capping agent, while hydroxyl groups from flavonoids prevented NP agglomeration [41]. The number of publications using such green synthesis methods is rapidly increasing, and their applications are expanding in various fields, especially in biomedicine, the pharmaceutical industry, photocatalysis, and functional device fabrication [13,18,42,43].

The plant-based green synthesis method described in this paper, using chamomile flower extracts, offers an environmentally friendly and sustainable alternative to conventional magnetite NP synthesis methods. Chamomile, an herb from the Asteraceae plant family, is found in various genera. The best-known chamomile species are German chamomile (*Matricaria chamomilla* L. syn: *Matricaria recutita* L.), Roman chamomile (*Chamaemelum nobile* L. All. syn: *Anthemis nobilis* L.), and Juhua (*Chrysanthemum morifolium* Ramat.) [44]. German chamomile is often preferred in commercially available chamomile tea as it tastes sweeter compared to Roman chamomile. To avoid confusion, *Matricaria recutita* L. or *Chamomilla recutita* is now the botanical name for chamomile, which belongs to the genus *Chamomilla* L. and the Asteraceae family. Chamomile is also a very grateful plant, as it grows almost like a weed in poor soils with limited water availability and is very easy to cultivate. The flowers are arranged in heads or a capitulum as an outer ring ray and inner disc flowers, a common feature of the Asteraceae family. *Matricaria chamomilla* L. has more biological effects than any other species. Chamomile is known for its positive effects on human health and has, therefore, been traditionally used for medicinal purposes for centuries. The biological activity of chamomile is primarily due to its numerous chemical constituents, which include around 50 flavonoids, 10 coumarins, 100 volatile oils, 70 terpenes (monoterpenes, sesquiterpenes, diterpenes, and triterpenes), 25 organic acids, 20 sterols and guaianolides, polysaccharides, and others. These compounds have anticancer, anti-infectious, anti-inflammatory, antithrombotic, antioxidant, hypolipidemic, hypoglycemic, antihypertensive, antidepressant, and neuroprotective effects. According to current knowledge about chamomile in the treatment of various diseases, its role as a medicine cannot be overlooked. Chamomile has been included in the pharmacopeias of 26 countries. Apart from the fact that it contains many identified substances with beneficial and therapeutic effects, it is a cosmopolitan plant—it is found on almost every continent [45,46,47,48,49,50].

While chamomile extract has been used to synthesize various types of NPs, including ZnO, MgO, MnO_2_ [51], and Ag [52], there is currently no report on the synthesis method for magnetite NPs using aqueous chamomile extract, and to the best of the author’s knowledge, this is the first such report. In line with the ongoing efforts to reduce environmental pollution, current research focuses on identifying materials that can replace chemicals in the conventional synthesis of NPs while achieving similar or better results. In this study, we aim to propose a novel and environmentally friendly approach for the synthesis of magnetite NPs. The microwave-assisted hydrothermal synthesis of Fe_3_O_4_ NPs was carried out in an alkaline medium using only FeCl_3_ as a precursor and aqueous chamomile extract. The aqueous chamomile extract serves a dual purpose: (i) the partial reduction of Fe^3+^ ions to Fe^2+^ ions at a precise molar ratio of 2:1, allowing for the formation of pure Fe_3_O_4_ and, thus, eliminating the need for an Fe(ii) precursor; and (ii) the biocoating, which could inhibit particle growth and, thus, create the magnetite particles non-toxic and biocompatible to human cells. Moreover, our promising cytotoxicity test results show that the synthesized magnetite NPs exhibit lower toxicity to healthy cells and higher toxicity to carcinoma cells. Those findings highlight magnetite NPs’ potential in biomedical applications.

## 2. Materials and Methods

### 2.1. Preparation of the Aqueous Chamomile Extract

The plant materials used in the present work are preprocessed flowers of chamomile, separated from any other plant tissues, such as roots, stems, or leaves (*Matricaria chamomilla*, commonly known as German chamomile) (Suban d.o.o., Strmec, Croatia). The dried flowers were crushed in a household blender, and 20 g of the crushed plant material was added to 200 mL of ultrapure water purified using the Millipore Simplicity 185 purification system (Millipore, Burlington, MA, USA) with a resistivity of 18.2 MΩ cm^−1^ at 25 °C. The mixture was heated to 60 °C and the temperature was kept constant for 20 min. The aqueous extract was then filtered and was ready for use after centrifugation (Beckman Avanti J-25, Indianapolis, IN, USA). 

### 2.2. Synthesis of Nanoparticles

**Scheme 1:**
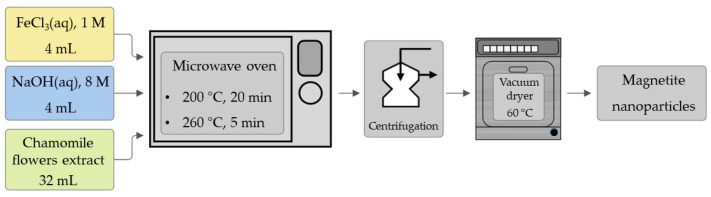
Schematic representation of magnetite nanoparticle synthesis.

The preparation of magnetite nanoparticles was performed according to Scheme 1, using anhydrous iron(iii) chloride, FeCl_3_ (p.a., Fluka, Charlotte, NC, USA) as only iron precursor. The alkalinity of the medium was ensured through the addition of sodium hydroxide solution, NaOH (p.a., T.T.T., Sveta Nedelja, Croatia). The two solutions mentioned above were prepared by accurately weighing and dissolving reagents in ultrapure water. The exact concentrations and volumes of solutions of iron(iii) chloride and sodium hydroxide were mixed with an aqueous chamomile extract in a Teflon vessel of a microwave oven (Milestone flexiWave SK15, Milestone, S.r.l., Sorisole, Italy) equipped with an ATC 400 sensor (Amphenol Advanced Sensors, St. Marys, TX, USA), in an exact volume of 40 mL, as summarized in Table 1.

The obtained precipitates were centrifuged, the mother liquor was isolated, and the pH of the solution was measured with a pH meter (MP220 Basic Mettler Toledo, Columbus, OH, USA). The precipitates were washed several times with ultrapure water and finally with ethanol. All precipitates were dried overnight at 60 °C in a vacuum dryer (Thermo Scientific, 3608–1CE, Waltham, MA, USA). For the comparison of magnetic properties, the magnetite NPs were also synthesized using a conventional chemical method utilizing Fe(iii) and Fe(ii) salts as precursors in a molar ratio of 2:1 (sample MagH), reported in our previous work [53]. The magnetite (sample MagH) particles were synthesized using the co-precipitation method, performed by mixing the weighted calculated mass of solid iron(II) chloride tetrahydrate (FeCl_2_·4H_2_O, pro analysis, 0.7952 g, VWR chemicals, Radnor, PA, USA), 8 mL of 1 M iron(III) chloride solution (prepared by dissolving anhydrous iron(III) chloride in water; FeCl_3_, pro analysis, Fluka, Charlotte, NC, USA), and 5.4 mL of concentrated ammonia (NH_3_, 25%, p.a., Gram mol, Zagreb, Croatia). The reaction mixture was then filled up to 40 mL with ultrapure water, transferred to a Teflon vessel, and heated up to 200 °C for 10 min in a microwave oven. After cooling, the precipitates were centrifuged and washed with ultrapure water and absolute ethanol [53].

### 2.3. Fourier-Transform Infrared Spectroscopy (FTIR)

The NPs were characterized with FTIR spectroscopy (Shimadzu IR Prestige-21, FTIR-8400S spectrophotometer, Kyoto, Japan) in the range of 400 to 4000 cm^−1^, using KBr pallets.

### 2.4. Powder X-ray Diffraction (PXRD) Measurements

Laboratory PXRD patterns were recorded at room temperature (RT) using the monochromatic Cu Kα X-ray source (*λ* = 1.5406 Å) on an Empyrean X-ray diffractometer (Malvern Panalytical Ltd. Malvern, Worcestershire, UK) over a 2*θ* range between 10° and 80° with a scan step of 0.053°. Structure refinements against powder diffraction data were performed with the Rietveld algorithm [54] using the X’Pert High Score Plus program (Version 4.1) [55]. The starting magnetite phase model was based on those of M. J. Fleet [56]. A pseudo-Voigt profile function and a polynomial background with up to four coefficients were applied to the structure refinements together with instrumental parameters (i.e., sample displacement and scaling factor), peak shape parameters, and lattice parameters. The quantitative phase analysis was carried out using the Hill and Howard formalism [57]. Crystallite size information was extracted from the phase-fitting method based on the change in profile widths compared to a standard sample. 

### 2.5. Magnetization Measurements

Magnetization measurements were performed on powder samples using an MPMS3 superconducting quantum interference device (SQUID) magnetometer (Quantum Design, San Diego, CA, USA). The temperature dependence of magnetization in a magnetic field of 100 Oe and 1 kOe was measured twice. First, the sample was cooled in a zero magnetic field (zero field cooled—ZFC), then the magnetic field was applied at the lowest temperature and the magnetization was measured while heating. In the second case, the sample was cooled in the same field (field cooled—FC) and the magnetization was measured while heating. The isothermal field dependence of the magnetization was measured in fields up to 7 T at several stable temperatures, where the magnetic field cyclically changed from +7 T to −7 T and back to +7 T to obtain a complete hysteresis loop. Due to the fine particles of the samples, they had to be embedded in paraffin to prevent the mechanical rotation of the particles in the magnetic field. The alternating current (AC) option of the same MPMS3 magnetometer was used to measure the temperature dependence of the AC magnetic susceptibility at several frequencies and at the zero-offset direct-current (DC) magnetic field.

### 2.6. Surface Morphology Imaging

Information on the morphology of the samples was obtained using a thermal field emission scanning electron microscope (FE-SEM), model JSM-7000F, manufactured by Jeol Ltd., Tokyo, Japan.

### 2.7. Cytotoxicity Tests

The cytotoxicity of magnetite NPs, namely samples S1 and S2, was studied using three different healthy human cell lines (HEK293, HaCaT, and HDF) and two carcinoma cell lines (HeLa and HCT116). The cells were grown in Dulbecco’s Modified Eagle Medium (DMEM) at 37 °C in a humidified environment with 5% CO_2_. Prior to the experiment, cells were detached from the surface of the fully confluent plates by adding 1 mL of a 0.05% trypsin–EDTA solution (Capricorn Scientific, Ebsdorfergrund, Germany) and resuspended in a DMEM medium (9 mL). The cells were counted using a handheld cell counter (Scepter, Merck, Darmstadt, Germany) and, if necessary, further diluted in DMEM to a concentration of 1 × 10^5^ cells mL^−1^. The samples S1 and S2 were prepared by suspending the NPs in sterile phosphate-buffered saline (PBS), 50 mg mL^−1^, and sonicating in an ultrasonic water bath at 37 °C for 1 h. The NPs were further diluted in DMEM to a concentration of 10 mg mL^−1^. The cytotoxicity experiment was performed in the microtiter plates containing 50 μL of the NPs in the twofold concentration range from 5.0 mg mL^−1^ to 4.9 µg mL^−1^ and 50 μL of cells (1 × 10^5^ cells mL^−1^) to each well. The control wells of the microtiter plates contained only NPs in the same concentration range, adjusted to the cell volume using pure DMEM. The plates were further incubated for 48 h under the same growth conditions. After this period, 20 µL of the 3-(4,5-dimethylthiazol-2-yl)-5-(3-carboxymethoxyphenyl)-2-(4-sulfophenyl)-2H-tetrazolium (MTS) reagent solution was added to each well according to the manufacturer’s instructions (CellTiter 96^®^ Aqueous One Solution Cell Proliferation Assay, Promega) and incubated for an additional three hours. The absorbance was then measured at 490 nm and the values obtained were corrected for the background from the nanoparticles, DMEM, and MTS. The IC_50_ values (concentration of NPs required for 50% inhibition) were determined from three independent measurements and calculated by plotting the concentration of nanoparticles against the percentage of live cells. The statistical analysis was performed using Student’s *t*-test in Excel comparing the mean IC_50_ values for S2 on healthy and carcinoma cell lines.

## 3. Results 

### 3.1. FTIR Spectroscopy Features

**Figure 1:**
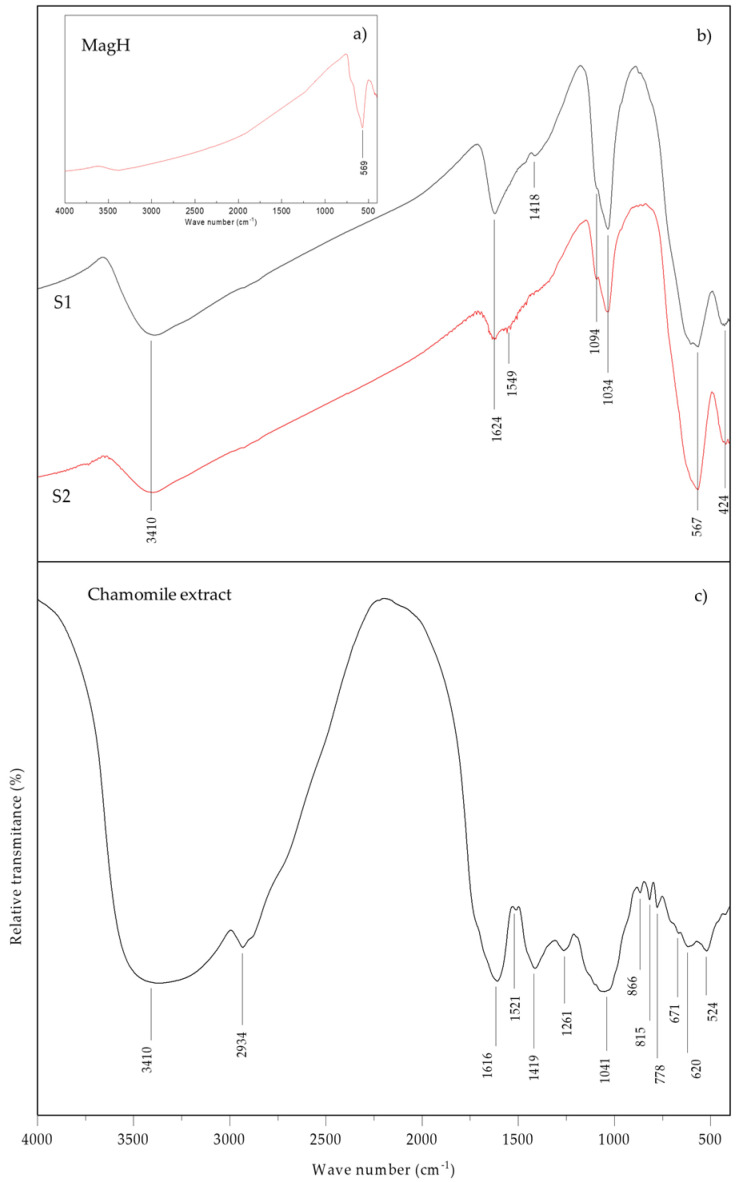
FTIR spectra of (**a**) sample MagH, (**b**) samples S1 and S2, and (**c**) dry residue of aqueous chamomile extract. Sample MagH is bare magnetite, prepared with the conventional method reported in the authors’ previous paper [53].

Figure 1b shows the FTIR spectra of samples S1 and S2, synthesized in the aqueous chamomile extract under different synthesis conditions (Table 1). These spectra show an intense band at 567 cm^−1^ as a result of Fe–O stretching vibration [58,59], confirming the formation of the magnetite phase in both S1 and S2. According to Figure 1c and the literature [52,60], absorptions at 3383, 2926, 1612, 1269, 1076, and 840 cm^−1^ of chamomile extract are in line with the O–H, C–H, C=C, C–O, and C–O–C stretching and O–H bending vibrations, respectively, which are the indications for the presence of polyphenols, flavonoids, and terpenoids in it. The IR band at ~1034 cm^−1^ in the spectra of samples S1 and S2 results from the C–O–C stretching vibrations and the band at ~1094 cm^−1^ can be attributed to the stretching vibration of the C–O bond, and O–H vibrations at 3410 cm^−1^, indicating the presence of polyphenols originating from the plant material of the aqueous chamomile extract. This confirms the formation of coated magnetite NPs. The FTIR spectra of sample MagH (Figure 1a) showed the same characteristic magnetite IR band at ~570 cm^−1^ but without the bands at ~1034 and ~1094 cm^−1^, as expected.

### 3.2. PXRD Characterization

It is clear from Table 2 that magnetite is the final product in both samples S1 and S2, regardless of the reaction temperature or time. Regarding the PXRD results [53] of sample MagH, this is consistent with the formation of magnetite by the conventional method.

**Figure 2:**
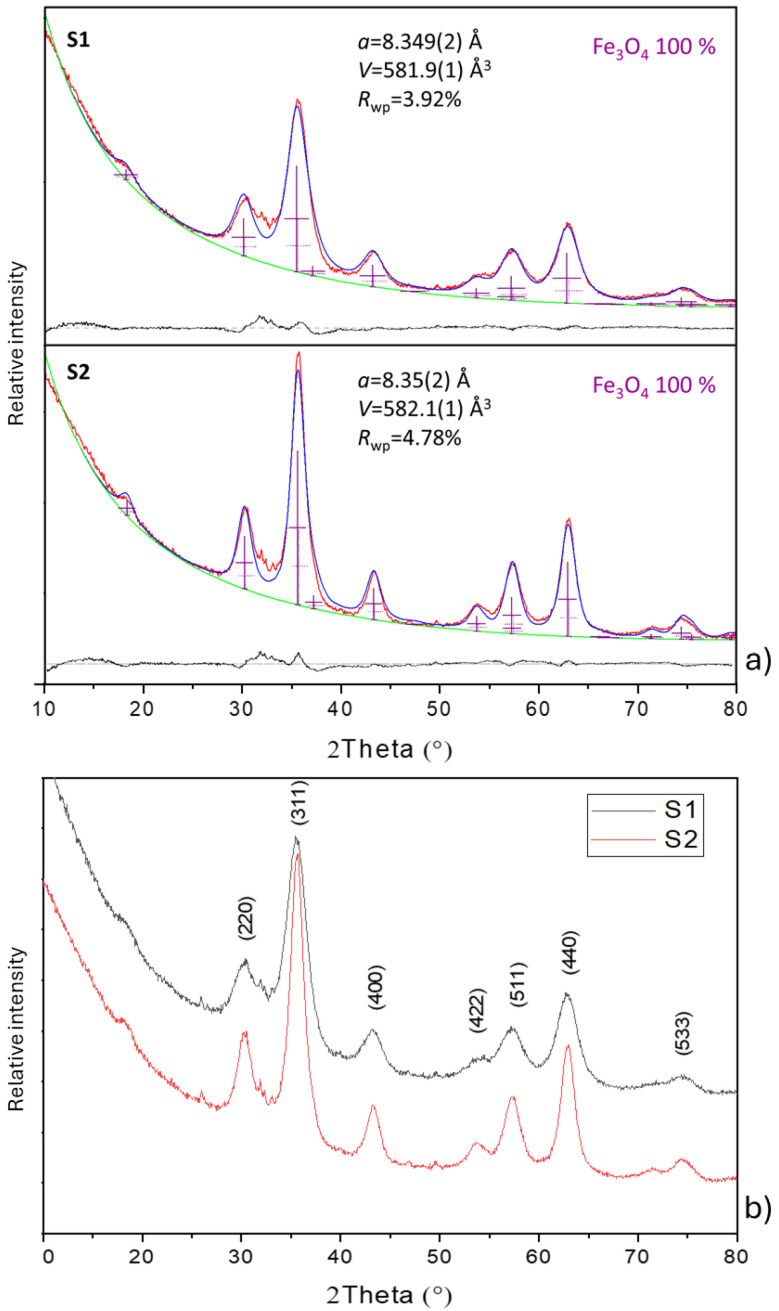
Comparison of (**a**) results obtained through Rietveld refinement against the PXRD data for samples S1 and S2, and (**b**) results of analysis performed by assigning reflection indices to the peaks in the diffraction data. In (**a**) the final observed and calculated powder diffraction profiles are shown by solid red and blue lines, respectively, while the background points are depicted by green line. The lower black solid lines show the difference profiles, while the inner purple tick marks indicate the reflection positions.

The PXRD patterns of samples S1 and S2 show rather low crystallinity; see Figure 2. Although microstructurally slightly different, with crystallites of ~8 and ~6 nm, the results of the structural refinement of such patterns show broad Bragg reflections corresponding exclusively to the formation of cubic magnetite (space group *Fd*-3*m*, No. 227), which can be well correlated with those observed in sample MagH [53].

The final observed and calculated powder diffraction profiles are shown by red and blue lines (Figure 2a), respectively. The fitted background contribution is represented by the lower green solid line, while the difference profile is represented by the black solid line. The purple inner markers indicate the position of the Bragg reflections corresponding to the Fe_3_O_4_ cubic phase.

An additional confirmation of obtained structural results was performed by assigning reflection indices (hkl) to the peaks in diffraction data. The reference pattern for magnetite was JCDPS Card 19-0629 and the indexed powder diffractogram can be observed in Figure 2b).

### 3.3. Magnetization Study

**Figure 3:**
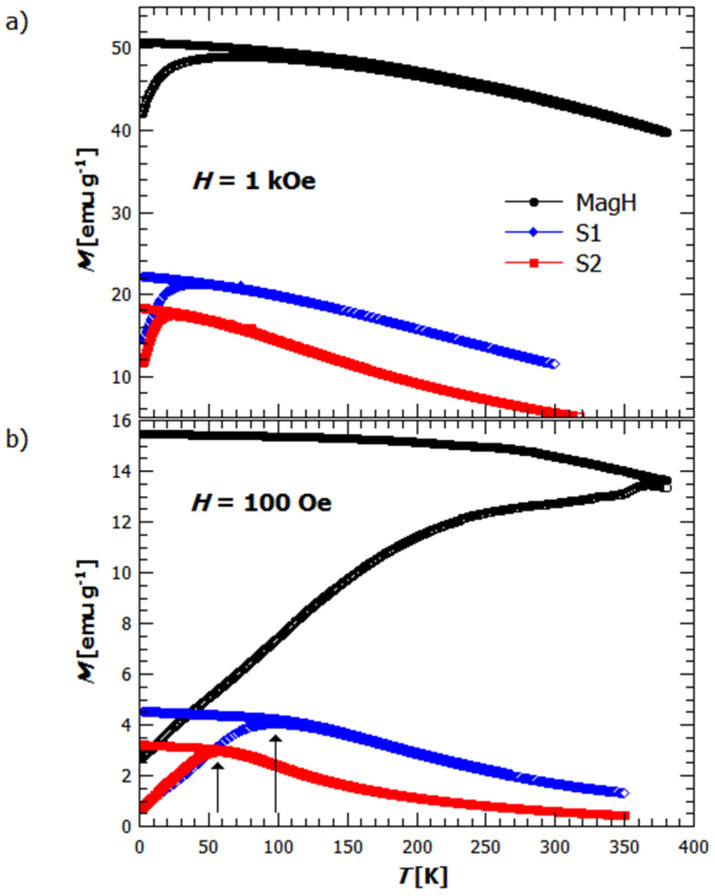
Comparison of the temperature dependence of the magnetization in the magnetic field of 1 kOe (**a**) and 100 Oe (**b**) for the samples MagH (black curve), S1 (blue curve), and S2 (red curve). The lower branches represent the ZFC curve, and the upper branches represent the FC curve. Arrows are eye guides to the maximum on the corresponding curves.

The temperature dependence of the magnetization *M*(*T*) of samples S1 and S2 and the sample MagH in a magnetic field *H* of 1 kOe and 100 Oe is shown in Figure 3. The zero field cooled (ZFC) curve is shown as lower branches and the field cooled (FC) curve as upper branches. The shape of the ZFC–FC curves in Figure 3a,b indicates the existence of nanoparticles with two well-defined temperature ranges: magnetic moment blocking below and superparamagnetic behavior above the blocking temperature. It is also evident, from the shape of the ZFC–FC curves for samples S1 and S2, that the particles are magnetically independent of temperature, in contrast to sample MagH, where a continuous increase in the ZFC curve is observed at 100 Oe (Figure 3b), probably due to the widely distributed particle sizes in combination with their magnetic interaction. The effective blocking temperature was determined from the maximum of the ZFC curve for S1 and S2 in the field of 100 Oe: *T*_B_ (S1) = 98 K and *T*_B_ (S2) = 57 K. 

**Figure 4:**
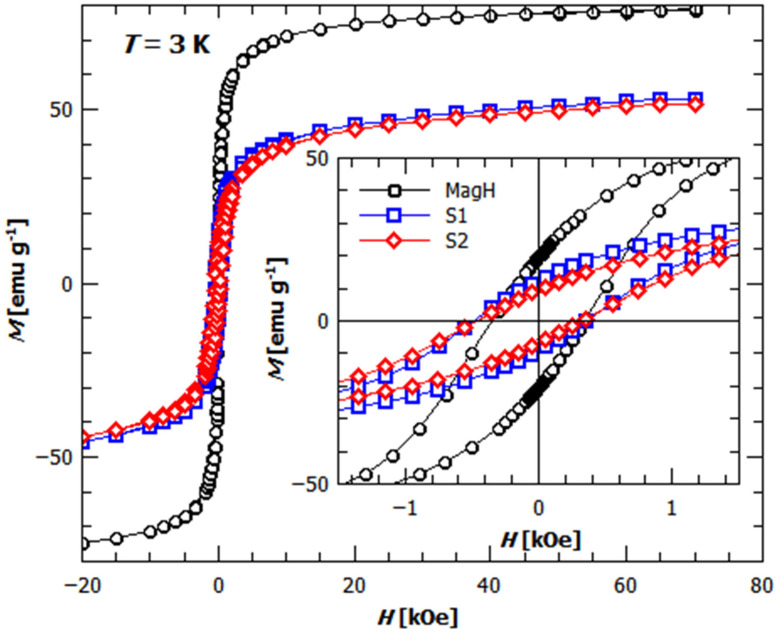
Magnetic field dependence of the magnetization at 3 K for the samples MagH (black circles), S1 (blue rectangles), and S2 (red diamonds). Inset: zoom around the zero magnetic field.

Figure 4 shows the field dependence of the magnetization for samples S1, S2, and MagH. The saturation magnetization (*M*_S_) or the value of magnetization at the highest applied field, 70 kOe, is 78.8 emu g^−1^, 53.1 emu g^−1,^ and 51.7 emu g^−1^ for samples MagH, S1, and S2, respectively. The theoretical saturated *M*_S_ value for bulk magnetite [61] is 92 emu g^−1^ and the sample MagH is closest to the theoretical value, as the sample MagH is a powder of bare magnetite nanoparticles, but it is still slightly lower due to the nanoparticulate shape and surface phenomena. 

The presence of the hysteresis curve (Figure 4, inset) at 3 K with an *H*_C_ around 0.4 kOe for all samples, together with the value of the saturation magnetization *M*_S_, indicates a ferrimagnetic ground state. However, due to the nanoparticulate microstructure, they behave like single-domain particles, resulting in an increased *H*_C_ in the blocked region, which is very different from the usual *H*_C_ in the bulk, being around 30 Oe or even lower [62,63,64]. 

**Figure 5:**
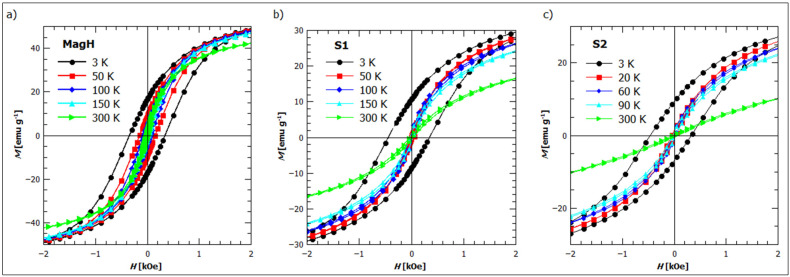
Magnetic field dependence of magnetization at different temperatures for the samples MagH (**a**), S1 (**b**), and S2 (**c**).

Figure 5 shows the magnetic field dependence of the magnetization *M*_S_(*H*) curves at different temperatures, from 3 K to room temperature for all samples. It can be seen that above the blocking temperature, the open hysteresis loops disappear, and the *M*(*H*) curves become reversible and show a shape typical of superparamagnets, although the magnetic ordering temperature of magnetite is up to 860 K [65]. According to the temperature dependence of the magnetization, the similarity of the coated particles in samples S1 and S2 compared to the slightly different behavior of the uncoated magnetite in sample MagH can be noticed. The steeper increase and faster saturation of sample MagH indicate that the full superparamagnetic state has not even been reached at room temperature, while the absence of *H*_C_ and a slower increase in magnetization with field in samples S1 and S2 are more indicative of their superparamagnetic state at temperatures above *T*_B_.

**Figure 6:**
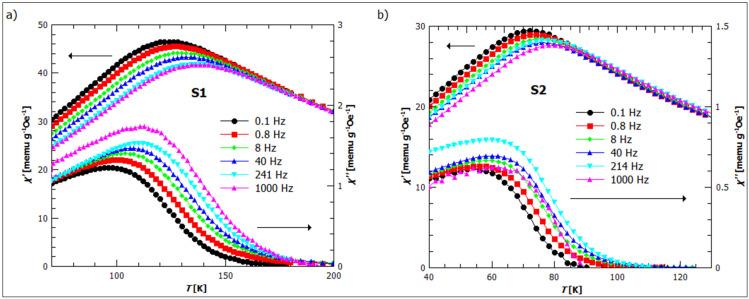
Temperature dependence of real *χ*′ and imaginary components *χ*″ of AC susceptibility measured for different frequencies on samples S1 (**a**) and S2 (**b**).

Figure 6 shows the temperature dependence of the real and imaginary components of AC susceptibility at different frequencies for samples S1 and S2. The higher temperature of the peak *T*_f_ in AC susceptibility for the sample S1 (*T*_f_ = 124–132 K) compared to the sample S2 (*T*_f_ = 72–76 K) in Figure 6a,b indicates larger particles in the sample S1, which is consistent with the results of the particle size from PXRD studies shown in Table 2. The AC magnetic susceptibility (*χ*) measurements for samples S1 and S2 show a frequency dependence typical of nanoparticles, with a broad and frequency-dependent maximum around *T*_B_ for the real in-phase component (*χ*′) and slightly lower temperatures for the imaginary out-of-phase component (*χ*″), reflecting the slow magnetization relaxation of the ensemble of single-domain particles. A noticeable shift in the peak values of real and imaginary susceptibility was observed (Figure 6) when the frequency was increased from 0.1 Hz to 1 kHz. The peak of susceptibility of the real component shifts towards higher temperatures and decreases with higher frequencies as fewer particles can switch their magnetization and more thermal energy is required to support the reorientation. The AC susceptibility measurements of the sample MagH showed no noticeable peaks of susceptibility in either the real or imaginary component. One possible reason for this is the agglomeration of the particles, which is accompanied by stronger interactions between the particles and blurs their superparamagnetic relaxation.

Table 3 summarizes some properties of investigated samples and will be explained in the Discussion to Section 4. 

### 3.4. Surface Morphology Imaging Features

**Figure 7:**
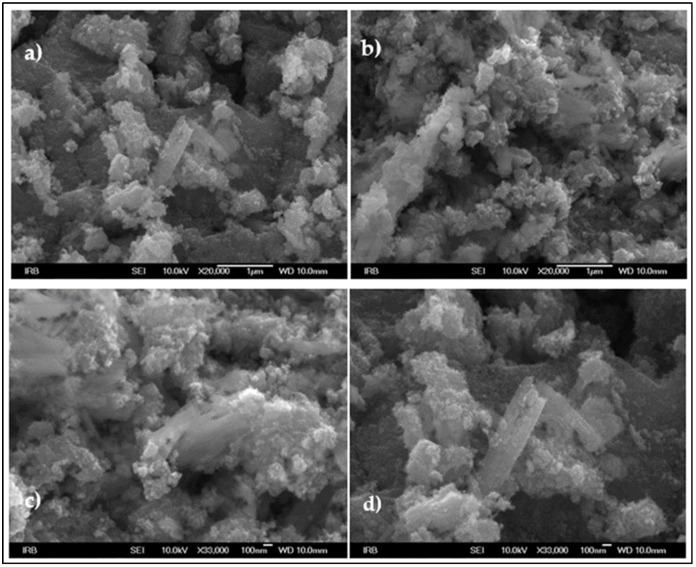
FE-SEM images of sample S1 at 20,000× magnification, (**a**,**b**), and S2 at 33,000× magnification, (**c**,**d**).

Figure 7 shows the results of the electron microscopic analysis of samples S1 and S2. The particles were imaged at magnifications of 20,000× and 33,000×. The magnetite NPs in sample S1 (Figure 7a,b) appear in irregularly shaped aggregates with an indication of rod-like formations probably due to the presence of plant material. Since particles in sample S2 were considerably smaller, they were observed at larger magnification, whereby the same rod-like morphology can be seen (Figure 7c,d). A similar morphology of magnetite NPs was previously reported for sample MagH [53] but without the presence of rod-like formations.

### 3.5. Cytotoxicity

The cytotoxicity of magnetic NPs, including unmodified magnetite (MagH), has been the subject of numerous studies. A swath of literature data regarding the toxicity of NPs, although controversial in some cases, indicates that magnetic NPs are essentially non-toxic and largely safe in biomedical applications. Based on these data, it appears that the surface coating, size, and concentration of the NPs influence the manifestation of toxicity, taking into account the exposure time and cell type [66].

Since a novel plant-based method for the synthesis of magnetic NPs was presented in this work, the resulting NPs were subjected to a cytotoxicity assay to prove that functionalization does not significantly alter their biological properties. Therefore, the in vitro cytotoxicity of S1 and S2 was determined with different cells in culture, namely healthy human cells of embryonic (HEK293), skin (HaCaT), and fibroblast (HDF) origin as well as with cervical (HeLa) and colon cancer cell lines (HCT116).

**Figure 8:**
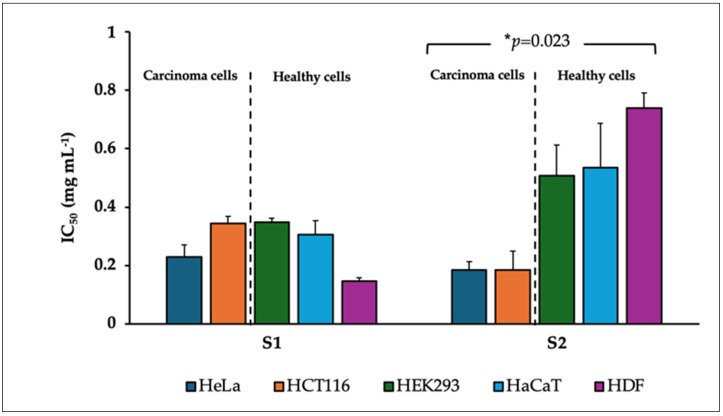
Cytotoxicity of S1 and S2 NPs on carcinoma (HeLa and HCT116) and healthy cell lines (HEK293, HaCaT, and HDF).

The results shown in Figure 8 are expressed as the inhibitory concentration (IC_50_), which is defined as the concentration at which 50% of the cells die. As can be seen in Figure 8, S1 showed a similar cytotoxicity profile across the cell panel, while S2 showed markedly different cytotoxicity for healthy cells (IC_50_~0.45–0.75 mg mL^−1^) compared to carcinoma cell lines (IC_50_~0.19 mg mL^−1^). Despite the apparent selectivity of S2, we must emphasize that the values obtained are still high in the physiological range (mg mL^−1^ concentration), where S2 is still considered non-toxic. These unexpected data may indicate a specific mode of action of S2 that makes it more toxic to carcinoma cells and opens up new possibilities that should be further investigated. 

## 4. Discussion

Targeted drug delivery is a technique that focuses on delivering drugs directly to a diseased part of the body while minimizing the IC effects of the drugs on other, healthy areas. As technology advances, so does the need for more effective drugs and delivery systems. Cancer is a disease that is considered the leading cause of death in most industrialized countries due to the limited effectiveness of conventional chemotherapy. The major shortcoming of conventional chemotherapy is the indiscriminate distribution of drugs in the body, which leads to suboptimal treatment outcomes and affects the overall well-being of the patient. Targeted drug delivery plays a crucial role in minimizing the side effects while maximizing the benefits of the drug. Metal-based NPs offer the potential to deliver drugs, but in most cases, the surface functionalization of metal-based NPs is required to efficiently encapsulate or bind drugs and control their release at the desired location [67]. Superparamagnetic iron oxide NPs, especially magnetite, are often used for targeted drug delivery due to their special magnetic behavior. 

Despite the existence of numerous synthesis methods, the formation mechanisms of magnetite NPs are still the subject of active investigations and ongoing discussions. Co-precipitation is a widely studied and simple technique for synthesizing magnetite NPs in which the experimental parameters play a crucial role in determining the properties of the final product [10,13]. In the co-precipitation method, the precursors Fe(iii) and Fe(ii) are precipitated simultaneously in a precise molar ratio of 2:1 in a pH range from 9 to 14. Several research papers [68,69,70,71,72,73,74,75,76] propose the following reactions for the mechanism of magnetite formation:

In the first step, iron(iii) and iron(ii) hydroxides are rapidly precipitated:

Fe^3+^(aq) + 3 OH^−^(aq) → Fe(OH)_3_(s)

Fe^2+^(aq) + 2 OH^−^(aq) → Fe(OH)_2_(s).

In the second step, iron(iii) hydroxide is converted to iron(iii) oxyhydroxide:

Fe(OH)_3_(s) → FeOOH(s) + H_2_O(l).

Finally, a solid-state reaction takes place between FeOOH and Fe(OH)_2_, which leads to the formation of magnetite:

2 FeOOH(s) + Fe(OH)_2_(s) → Fe_3_O_4_(s) + 2 H_2_O(l).

The overall reaction can be summarized as follows:

2 Fe^3+^(aq) + Fe^2+^(aq) + 8 OH^−^(aq) → 2 Fe(OH)_3_(s) + Fe(OH)_2_(s) → Fe_3_O_4_(s) + 4 H_2_O(l).

The influence of plants or plant metabolites on the synthesis of magnetite NP for targeted applications could be highly desirable and useful as they can act as reducing, stabilizing, and capping agents. The aqueous extract of chamomile flowers is known for its diverse pharmaceutical and medicinal applications. More specifically, German chamomile contains bioactive components such as terpenoids (α-bisabolol, chamazulene, and sesquiterpenes), coumarins (umbelliferone), flavonoids (luteolin, apigenin, and quercetin), spiroethers (dicycloethers), and other components such as tannins, glucosides (anthemic acid), choline, polysaccharides, and phytoestrogens. These compounds have been found to stabilize and passivate the reduced NPs [18,47].

In this study, we successfully optimized a microwave–hydrothermal method to produce magnetite NPs. Our approach involved two simplified variants of experiment, in both cases using a single starting material, an iron(iii) precursor: 0.1 M FeCl_3_ dissolved in an aqueous chamomile flower extract. One experiment setup included heating at 200 °C for 20 min (S1) and the other at 260 °C for 5 min (S2). The same method was used in our previous work with the same experimental parameters but in pure water [77], where we obtained a mixture of goethite and hematite at 200 °C for 20 min while we observed the formation of monophasic hematite at 260 °C for 5 min.

**Scheme 2:**
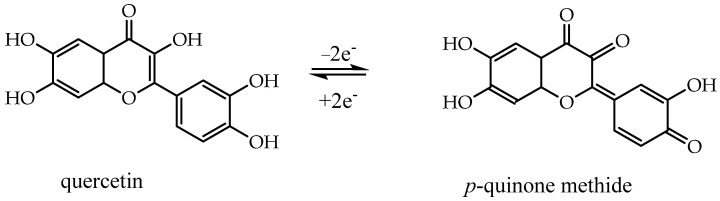
An example of the formation of a quinoid intermediate.

It is known that some flavonoids have the ability to undergo enzymatic and/or non-enzymatic oxidation reactions that convert them into electrophilic quinoid species (e.g., semiquinones and quinone methides) and/or certain reactive oxygen species (ROS) [78,79,80]. It is also known that flavonoids bearing two or more hydroxyl groups in their B ring are recognized to be more prone to form quinoid intermediates, according to Scheme 2.

The latter species could be involved in the interaction of some specific flavonoids (i.e., diphenols) with (i) certain ROS (e.g., superoxide anions, hydroxyl, and peroxyl radicals), as the flavonoids are converted to phenoxyl radicals and possibly to quinoid species after their scavenging or reduction; (ii) catalytic concentrations of some redox-active transition metals, which in their reduced state (e.g., Cu^+^ or Fe^2+^) and in the presence of oxygen generate superoxide anions; and (iii) certain metalloenzymes (e.g., peroxidases, tyrosinases, and oxidoreductases) capable of catalyzing their oxidation, leading to the formation of semiquinones and quinones. Phenolic compounds such as apigenin are also known to be bioactive compounds in chamomile extracts. 

**Scheme 3:**
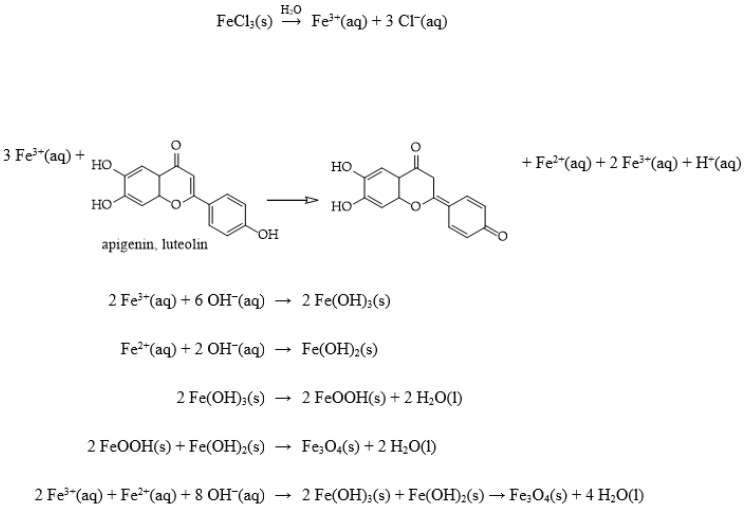
Proposed mechanism of oxidation-reduction in the synthesis of magnetite.

Scheme 3 shows our proposed mechanism and suggests that oxidation-reduction plays a role in the synthesis of magnetite in chamomile extracts, as opposed to goethite or hematite, as shown in our previous work [77]. We concluded that chamomile extract is an appropriate medium for the reduction of Fe^3+^ to Fe^2+^ because the reduction achieves a required 2:1 molar ratio of Fe^3+^ and Fe^2+^ for magnetite formation. Indeed, the single-phase PXRD patterns of S1 and S2 show a cubic symmetric crystal structure of magnetite with space group *Fd-3m*, as shown through PXRD analysis and Rietveld refinement (Figure 2a and Table 2). The magnetic properties, particle size, and biocoating of magnetite play a crucial role in the biomedical applications of magnetite. Superparamagnetic behavior is desirable because when exposed to an external magnetic field, these nanoparticles exhibit a remarkable response that allows them to be directed and concentrated with exceptional precision to specific regions or to target diseased areas in the body [81].

Regarding the particle size, as described in the introduction, too small and uncoated NPs could penetrate physical barriers and come into contact with intracellular compartments, which could lead to cell damage or destruction. Therefore, a suitable capping agent could potentially reduce toxic effects by forming a coating on the surface of the NPs that prevents direct contact between the NPs and cellular components, thus minimizing the risk of cell damage. Since particle size is strongly influenced by the temperature and reaction time in a synthesis process, we conducted two series of experiments with different parameters. The reaction mixture of sample S1 was exposed to a lower temperature of 200 °C with a longer reaction time of 20 min, while the reaction mixture of sample S2 was exposed to a higher temperature of 260 °C with a shorter reaction time of 5 min. By adjusting the temperature and reaction time, we attempted to promote either nucleation or crystal growth in the synthesis process. Higher temperatures promote faster nucleation, whereas a shorter time leads to the formation of smaller particles, which is, therefore, expected for sample S2. Conversely, lower temperatures promote slower nucleation, and prolonged reaction time leads to the formation of larger particles, as expected for sample S1. As assumed, slightly larger crystallites are observed in sample S1 than in sample S2, as shown in Table 2. However, both samples, S1 and S2, exhibit low crystallinity, as can be seen from the Rietveld structure refinements in Figure 2a which is most likely due to the chamomile extract in the synthesis process.

A comparison of the magnetic properties between the magnetite particles of samples S1 and S2 prepared via the chamomile extract-mediated synthesis and the sample MagH was performed and yielded interesting results. The magnetization value of samples S1 and S2 compared to the sample MagH, as shown in Figure 3 and Figure 4, is almost twice as small compared to the magnetite of the sample MagH. This is due to the slightly lower percentage of magnetite in the total mass of the powder sample, as the magnetite nanoparticles of S1 and S2 are coated. From the ratio of the saturation values of the bare magnetite in sample MagH and the coated magnetite in S1 and S2, we can determine the approximate amount of coating in samples S1 and S2, namely 32.6% for S1 and 34.4% for S2. Figure 3b shows that the magnetite particles in the sample MagH have a non-uniform size distribution since the volume of each particle is one of the main factors determining the blocking temperature. Furthermore, the ZFC–FC splitting for the sample MagH did not occur at the temperature at which the ZFC curve has its maximum but well above this point so that the determined blocking temperature is actually an effective value at which most of the particles are blocked. The effective blocking temperatures for samples S1 and S2 shown in Figure 3b, *T*_B_ (S1) = 98 K and *T*_B_ (S2) = 57 K, correspond to the blocking temperatures of the particles that contribute most to the magnetization. Since the magnetite nanoparticles in samples S1 and S2 are capped and the organic coating shows no magnetic response (except diamagnetic, which is negligible compared to the ferrimagnetic magnetite), these temperatures are lower than for the uncoated particles in sample MagH, the sample with the higher percentage of the magnetite in the total mass of the sample. The higher blocking temperature *T*_B_ of sample S1 compared to S2 is consistent with the observation of smaller particle sizes in S2. A simple analysis uses the following equation:

$$K\times V=25\times k_{B}\times T_{B}$$

with the volumes *V* calculated from the particle diameters in Table 2 yields a density of magnetic anisotropy energy *K* that is 2 orders of magnitude smaller than that found in the literature [82,83], which cannot be attributed to a small correction of the surface anisotropy terms, or interactions. Therefore, we calculated the expected diameters assuming that the energy density of magnetic anisotropy [61] is 5000 J m^−3^. The values for the magnetic diameters (*d*_mag_) obtained using the above formula for S1 and S2 are 10.8 nm and 9.0 nm, respectively (Table 3). These magnetic diameters are only slightly larger than those in Table 2 due to the effect of agglomeration, suggesting good agreement with sizes obtained with the X-ray diffraction. In fact, their interaction in agglomerated particles, which are still relatively small, leads to second-order corrections in the calculated magnetic parameters, since there are only several particles in agglomerate. However, without taking these smaller nanoparticles into account (smaller than what is visible with electron microscopy and X-ray diffraction), it is not possible to explain the observed magnetic behavior. Furthermore, it cannot be assumed that the observed particles on the SEM images are single-coated magnetic nanoparticles. Based on the number of non-magnetic shells around the magnetic cores determined above, the relative amount of magnetite to the mass of the sample is around 67%. On the other hand, the sizes of the magnetic cores were determined from magnetic measurements and the above-mentioned model of blocking, leading to the conclusion that the size of the particles is much smaller than observed with the SEM. Therefore, the coating (33% of the mass) around the small magnetic cores is not consistent with the nature of the observed grains as single particles but suggests that the SEM images actually show agglomerates of several magnetic nanoparticles coated with an organic shell.

Figure 6 shows that the AC susceptibility peaks shift towards higher temperatures for samples S1 and S2. It also points out the reduction in magnitude with higher frequencies as fewer particles can switch their magnetization and more thermal energy is required to help the reorientation. The AC susceptibility measurements of the sample MagH showed no noticeable peaks in either the real or imaginary components of susceptibility. One possible reason for this is the agglomeration of the particles, which is accompanied by stronger interactions between the particles, blurring their superparamagnetic relaxation.

From the data in Figure 3, *T*_irr_ (S1) = 180 K and *T*_irr_ (S2) = 100 K, it can be seen that the presence of larger particles in sample S1 causes the ZFC–FC to split at much higher irreversibility temperatures. For comparison, the narrower size distribution of the magnetite nanoparticles is reflected in *T*_irr_, which is quite close to *T*_B_ [84]. When measured in a higher magnetic field, *T*_irr_ and *T*_B_ are closer to each other, as can be seen in Figure 3a, since most of the particles are magnetized to their equilibrium state. Furthermore, the AC magnetic susceptibility (see Figure 6) confirms that the particles are larger in sample S1 than in S2, as the peak in the S1 sample occurs at a higher temperature than in sample S2, with the same conclusion as in the direct-current (DC) magnetization measurements. The frequency dependence of the imaginary susceptibility peak from Figure 6 can also be used to calculate the activation energy *E* for the thermal reversal of magnetic moments of the magnetic entities and time constant $\tau_{0}$ by fitting the standard Arrhenius law [83]. For S1 we obtain:

$$E/k_{B}=6480\;K\;and\;\tau_{0}=2.8\times 10^{-29}\;s\;\mathrm{and}\;\mathrm{for}\;S2\;E/k_{B}=3770\;K\;\mathrm{and}\;\tau_{0}=2.9\times 10^{-30}\;s.$$

This approach is an additionally useful argument because the relative shift of the peak per frequency decade Δ*T*_f_/*T*_f_Δlog_10_*f* is larger than 0.03, which argues for superparamagnetism and excludes eventual spin-glass behavior [85]. The obtained activation energy for S1 and S2 is 2.6 times larger than the corresponding barrier heights of the single nanoparticle K×V obtained from the quasi-static ZFC–FC measurements and determined from blocking temperatures. This relation is an additional argument for interaction between particles [83] and is consistent for both samples, S1 and S2, showing again that magnetic entities, which are excited to thermal reversal with AC field, are actually agglomerates of several particles, which is in accordance with the above static magnetic results. Quite opposite, no such frequency dependence was observed for the bare magnetite (MagH), as no peak in the susceptibility was observed up to the highest temperature of 400 K, again pointing to the agglomeration of the nanoparticles, where no superparamagnetism and blocking occurred due to the interactions of the particles. Evidently, the organic coating played an important role in the observation of these signatures of independent nanomagnets. 

In the magnetic sense, the main role of the chamomile extract is to prevent the overall agglomeration that apparently occurred in the sample MagH. Indeed, sample MagH cannot be described as a collection of relatively independent magnetic nanoparticles, as is the case for samples S1 and S2. This is due to the lack of a well-defined blocking temperature as well as the much steeper hysteresis of the particles, all of which indicate an overall agglomeration and a strong magnetic interaction between neighboring nanocrystallites. 

The cytotoxicity of the newly synthesized NPs, namely S1 and S2, was not compared with iron oxide nanoparticles (IONs), in particular magnetite (MagH), as IONs have been shown to be essentially non-toxic in most studies and are generally considered safe in biomedical applications [66]. Since a novel plant-based method for the synthesis of magnetic NPs was presented in this paper, the resulting NPs were subjected to a cytotoxicity test to prove that the functionalization did not significantly alter their biological properties, or simply put, that they did not become toxic through the modification process. Like MagH, the new S1 and S2 did not show physiologically relevant toxicity as their IC_50_ values were in high mg mL^−1^ concentrations. However, we observed a different cytotoxic behavior of S1 and S2. The S1 NPs showed a similar cytotoxicity profile across the cell panel, while S2 showed markedly different cytotoxicity to healthy cells (IC_50_~0.45–0.75 mg mL^−1^) compared to carcinoma cell lines (IC_50_~0.19 mg mL^−1^). Despite the apparently greater toxicity of S1 toward carcinoma cell lines, the values obtained are in the range of those published for MagH (100–1600 μg mL^−1^) [86]. Not only did S1 and S2 show a different cytotoxicity profile but the apparently stronger toxic effect of S2 on carcinoma cell lines also proved to be statistically relevant.

Particles with nanoscale size and a suitable capping agent efficiently minimize the risk of cell damage in healthy cells, while they exhibit surprising toxicity only for cancer cells. The aqueous extract of chamomile flowers, known for its extensive pharmaceutical and medicinal applications, probably contributes to this effect. By optimizing the reaction conditions and using aqueous chamomile flower extract as a novel medium, we have succeeded in producing magnetite NPs that are harmless to healthy cells and toxic to carcinoma cells using a more environmentally friendly and simplified microwave–hydrothermal method.

## 5. Conclusions

This work shows that the precipitation parameters play a crucial role in the synthesis and coating of magnetite NPs. From the magnetic point of view, plant-mediated coating played an important role in expressing the signatures of independent nanomagnets, where, contrary to the bare magnetite, the coated nanoparticles showed the superparamagnetism and dynamical nature of the magnetic moment blocking. Such a coating not only offers other functionalities for the application but also the possibility to tune the distances between the magnetic nanoparticles and further investigate the influence of controlled interactions on their magnetic behavior. Understanding the role of the plant material components provides valuable insights for developing efficient synthesis methods tailoring the properties of magnetite NPs for various applications.

### Future Work

Our next task will be to understand the mechanism and to precisely identify the active components in the extracts used and their role in the synthesis process. This is important in order to simplify the synthesis route by focusing on one or two components that play a key role in the reduction of metal cations and provide stabilization and coating. Identifying these components is crucial in designing magnetite NPs with improved properties. Our findings are encouraging for further investigations, especially for the upcoming design of NPs with desired properties for specific applications.

## Figures and Tables

**Table 1:** Experimental conditions for the synthesis of magnetite NPs.

Sample	FeCl_3_, 1 M (mL)	NaOH, 8 M (mL)	Aqueous Chamomile Flowers Extract (mL)	Temperature (°C)	Reaction Time (min)
S1	4	4	32	200	20
S2	4	4	32	260	5

**Table 2:** The PXRD analyses results for the synthesized products. Standard deviations are given in parentheses.

Sample	Unit Cell Metrics (Å) Fe_3_O_4_ (s.g. *Fd*-3*m*)	Crystallite Size (Å)	Phase Fraction (%)	*R*_wp_ (%)
S1	*a* = 8.349 (2)	76.2 (1)	100	3.92
S2	*a* = 8.35 (2)	57.4 (1)	100	4.78

**Table 3:** Results of the magnetization measurements.

Sample	Size /nm	*T*_B_ /K	*T*_irr_ /K	*E*_A_ * /J m^−3^	*M*_s_ /emu g^−1^	*H*_C_/Oe	*T*_f1_ ^1^/K	*T*_f2_ ^2^/K	*PHI* ^3^	*E*_Arrh_/K
MagH	-	-	-	-	78.8 ± 0.1	350 ± 10	-	-	-	-
S1	79.4	98 ± 1	180 ± 3	16.1 ± 0.4	53.1 ± 0.1	400 ± 10	124/98 ± 1	132/107 ± 1	0.036 ± 0.003	6480 ± 80
S2	61.5	57 ± 1	100 ± 3	20.2 ± 0.6	51.7 ± 0.1	400 ± 10	72/56 ± 1	76/60 ± 1	0.03 ± 0.003	3770 ± 50

* *E*_A_ obtained from the formula 25*k*_B_*T*_B_/*V*; ^1^—temperature at the maximum value of susceptibility at frequency 0.1 Hz for Re/Im curves; ^2^—temperature at the maximum value of susceptibility at frequency 40 Hz for Re/Im curves; ^3^—PHI = Δ*T*_f_/*T*_f_Δlog_10_*f.*

## Data Availability

Data are contained within the article.
